# Editorial

**Published:** 1865-08

**Authors:** 


### Editorial.

EXPLANATION.—To prevent the necessity of dividing the valuable report of Dr. PRINCE, we have added 48 pages to the present number of the EXAMINER, and thereby included it entire in this issue. We have several communications on hand, which will appear in the next number, together with the usual notices of new books.

SURGEONS FOR THE ARMY.—In the July number of the EXAMINER, was a notice from Surgeon WOODWORTH, at Louisville, Ky., asking for assistant-surgeons, to be employed by contract. Owing to orders from the War Department, making changes in the number and location of the soldiers in that district, the notice has been recalled, there being no further need of assistance in that quarter at present.

IMMORAL MEDICAL ADVERTISEMENTS.—We have just received the Proceedings of a recent meeting of the Fox River Valley Medical Association, and insert them below. The preamble and resolutions in relation to the criminality of a certain species of newspaper advertisements, we fully approve.

We know of no more monstrous system of public swindling on the one hand, and of pandering to vice on the other, than is practiced by newspaper proprietors, in publishing pretended preventives of pregnancy, cures for *private* diseases, and whole columns of obscene advertisements about Quacks who pretend to cure venereal diseases. That the proprietors of newspapers are *particeps criminis*, and morally responsible for all the consequences resulting from such advertisements, is too obvious to every intelligent mind, to need either argument or illustration.

### PROCEEDINGS OF THE FOX RIVER VALLEY MEDICAL ASSOCIATION.

The Association met in the Common Council Room, in the city of Elgin, on Monday afternoon, July 3d, 1865.

The President, Dr Tefft, in the chair.

Present Drs. Tefft, Howell, Burdick, Woodworth, Tyler, Jassoy, Bartels, Hawley, Eddy, Reed, and Young.

Minutes of last meeting read and approved.

Dr. Howell proposed Dr. John Jassoy for membership.

On motion of Dr. Young, Dr. Jassoy was elected, when he came forward, paid the admission fee, signed the Constitution, and duly became a member of the Association.

Dr. Hawley announced that the Chicago *Republican* refused to publish *criminal* quack advertisements, and that the Ohio State Medical Association had passed resolutions commending its course, and moved that a committee be appointed by the chair to draft resolutions expressive of the sense of this Association. The President appointed Drs. Hawley, Howell, and Woodworth such committee.

Dr. Tefft read an interesting paper giving an account of a case of cancer of the stomach that had occurred in his practice recently.

Dr. Burdick reported a case of malignant tumor of the jaw that was successfully removed by an operation.

Dr. Young reported an interesting case of abscess of the lungs, in a child. The child recovered after a protracted illness.

Dr. Tefft presented a patient with inflammation and swelling of the inferior extremities of long standing. Quite a discussion ensued as to the pathology of the case, which was participated in by Drs. Howell, Jassoy, Tefft, Hawley, Tyler, and Young.

The committee on resolutions reported as follows:—

*Whereas*, The admission by the public press of advertisements of an immoral character, inviting the commission of crime by producing abortion and preventing pregnancy, for a consideration, in the shape of pills, potions, drops, etc., is terribly demoralizing and destructive of health and life, we therefore hail with pleasure the promise of the Chicago *Republican* that they will not admit advertisements of that character into their paper; therefore,—

*Resolved*, That the moral integrity and noble stand of its proprietors in excluding this class of advertisements from its columns, merits our highest approbation, and we hereby pledge ourselves to take and recommend only such papers as best conform with the principles of morality on this subject, believing that we, as guardians of the public health and conservators of public morals, cannot too strongly reprobate the publication in religious papers of advertisements, (for money,) the object of which is clearly the prevention of pregnancy or the commission of child murder, to commit crime, pander to lust, and that too frequently at the expence of the lives of the mothers, as well as their offspring.

*Resolved*, That the wide spread influence of a certain looseness of sentiment on this subject should be matter of great alarm to the philanthropic physician, as well as the parent, and that every public print that will pander to this species of crime is wickedly suicidal of the best interest of our race, and unworthy to be received into family circles or the patronage of honest men.

*Resolved*, That we deem those druggists who advertise and sell such preparations as *particeps criminis*; we hereby earnestly recommend them to discard them from their stocks and refuse to furnish them.

The report was accepted and adopted, and the Secretary instructed to publish them in the Chicago Medical Journals, Chicago *Republican*, and Aurora *Beacon*.

Dr. Burdick moved that a vote of thanks be tendered the proprietors of the Aurora *Beacon* for their generosity and promptness in publishing the proceedings of our Association. The resolution was adopted unanimously.

On motion of Dr. Burdick, the Association adjourned to meet in the Common Council Room in the city of Aurora, on the first Monday of October next, at 1 o'clock, p.m.

JOS. TEFFT, M.D., Pres.

D. W. YOUNG, M.D., Sec.

### LETTER FROM PROF. JOHNSON.

PARIS, June 15, 1865.

DEAR DOCTOR:—I have for more than two months been in Italy, enjoying its natural beauty, its rich treasures of art, and becoming, to some extent, acquainted with the state of medical science and practice in this land of wonders.

Among the ancient statuary, I have looked with especial satisfaction upon the divine Esculapius, and the busts of Hippocrates and Galen. Esculapius, indeed, is among the most common of ancient statues, and is always represented with a dignity of form, and a benevolence and intelligence of expression, clearly indicating the public respect for the art over which he presided. Indeed, the statues of Jupiter and Esculapius more nearly resemble each other than any two sculptures with which I am acquainted. The heads of Hippocrates all have the same general expression; the features all indicate, in a remarkable degree, intelligence, candor, and benevolence; the face, though in stone, reveals a heart warm and gentle, one that a child might love, and that a philosopher must respect. The busts are, most likely, all copies of some older original, and, perhaps, a faithful portrait.

At Rome, I visited what was once the home of Galen. In the evening twilight I walked along the *via sacra*, and, between the capitol and the colosseum, stood before the shrine that, for 1400 years, gave law and authority to medicine. I looked up and down the deserted street, along which the ancient Romans, with trembling steps, crowded to his doorway. On either hand, temples in ruins; behind me, the prostrate columns of the Roman forum, and, still farther, the palaces of the Cæsars, a shapeless mass, from which the owl was gloomily calling to the ghosts of the dead old years; all around me, grinning skeletons with which time has made sad havoc, bones broken, parts lost, symmetry destroyed—death, ruin, forgetfulness. The great man, however, who lived here in the days of Rome's glory, when all was beautiful and grand, did something better than to build palaces of stone, he brought to light great truths more imperishable than marble, and out of them constructed a temple, whose altars are still beaming and burning with the fires of science and the light of human sympathy and charity. The very bust of Galen beams with intellectual life and vigor, with, perhaps, less benevolence than Hippocrates, there is in the features something more suggestive of creation, of invention, of theory. One would say that the Greek was, perhaps, the best observer, and was satisfied with what he saw, while the Roman was the most scientific, constantly asking for the causes of things.

While at Naples, I visited Pompeii and the *Museo Internazional*, which contains, among many other objects illustrating the daily life of the ancient Romans, the remains of an apothecary's shop, and a large number of surgical instruments. I noticed *probes, forceps, sounds, catheters, scalpels, and a speculum ani, and a speculum uteri,* these last not unlike those in use at the present day. Among the frescoes of Pompeii, is a group representing a surgeon closing with stitches a large wound. The incision is over the region of the femoral artery. The patient is standing, the mouth firmly closed, the features indicating suffering and resignation. The wife looks on with anxious sympathy, while a child, of apparently a dozen years, is clinging to the father's hand in an agony of grief. The surgeon, in a half kneeling posture, is holding the needle with the forceps. The expression of his countenance is calm, confident, and intelligent, at once commanding respect. The whole painting is excellently well-preserved.

In my wanderings in Italy I visited Pisa, and Padua, and Bologna, so intimately associated with the revival of medical studies after the long night of ages. It was at Bologna, as you will remember, that anatomy was first taught practically. It was there also, that Galvani, in 1789, discovered that form of electricity that bears his name.

At Florence, I saw the celebrated Museum of Natural History. The illustrations of comparative and human anatomy are especially valuable. I visited, also, the hospital of *St. Maria Nuova.* This institution contains 1000 beds, and is open to all classes, to the poor, gratis, while there are rooms for those who are able to pay, varying in price from two to forty francs a week. *Post mortem* examinations are practiced, and the material for the study of normal and pathological anatomy is abundant. The medical department of the University of Pisa is located at Florence, and is connected with this hospital. The anatomical museum, though not large, is valuable. Among its curiosities are the preparations made by Segate, and described by an American surgeon as petrifactions. The skin is very perfectly preserved, retaining nearly its natural color; other tissues are somewhat changed, but do not decay. There is, in the museum, a table, in mosaic, made of the tissues of the different organs, such as the liver, lungs, kidneys, spleen, &c. The process seems to have been rather a *cornification* than a *petrifaction*. The art was a secret, and perished with the discoverer. The rooms for the accommodation of the medical school are in the hospital building, and would, with us, be considered shabby. The means of teaching, however, are excellent, and the curriculum of study very full and extensive. The doctorate is reached after seven years pupilage, five of which may be spent at Pisa, in the study of the more fundamental branches, and two in the hospital at Florence. The medical department has ten professors, and instruction is given throughout the year. I called on Prof. FELIPPO PACINI, the celebrated anatomist. He is a noble looking man, perhaps fifty years old, and an untiring student. I found him in his study, with his microscope, at work over a pathological specimen. He spoke kindly of America, asked after our scientific men, and expressed a sympathy with us in our political struggles, and especially in our national bereavement. He has a very valuable cabinet of microscopic specimens, and, as a souvenir of our interview, gave one of his preparations.

There are several medical schools in the Italian kingdom, and one at Rome. There are also many ripe scholars and men of fine scientific attainments, and yet, from all I can learn, I am satisfied that the *practice* of medicine and surgery is inferior to that of Northern Europe or America. There is a wonderful respect here for authority. The skeleton hands of the dead old masters still write prescriptions for Italian women and children. There is another fact that perhaps, to some extent, affects both medical and surgical practice. Human life is of too little value, and patients and their attendants are careless, not only in consulting a physician, but also in following his advice. Among the lower classes, in some parts of Italy, fractures and dislocations are left to nature, and the services of the surgeon are seldom or never called in requisition to correct deformities. With us, human muscles and the organs of sense, if not in themselves wealth, are certainly the means of securing a competency, but here, their absence, if not absolute riches, at least secures admission to a profitable and not dishonorable profession. A half-blind and, apparently, idiotic cripple makes, generally, a very prosperous, thrifty beggar. Hans Anderson's "Uncle Peppo" is by no means confined to Rome, and there are vague hints, that many an orphan child has suffered sad tortures, to qualify it for the "Spanish steps."*

The sanitary condition of the Italian cities, especially in the South, is not good. Naples, with a population of 500,000, situated on one of the most beautiful bays in the world, with its wonderful treasures of art and its environs, so rich in historical associations; its mountains, its promontories, and its islands; its sky, so clear that the very sun and moon and stars seem full of joy, as they go swimming through its liquid depths; its sea of Tyrrhian blue lighting, as with flames of burning sulphur, the caverns of its milky coast. Naples, with all these attractions, would be a most delightful place in which to spend one's days, but for "the pestilence that walketh at noonday." It is a filthy city. Its most attractive street is the hotbed of fevers, the type of which is always typhus or typhoid, and frequently of the most malignant form. Many a traveller who has spent a most delightful winter in the enjoyment of the rich physical beauty of this unequalled landscape, has fallen at Rome or at Florence, a victim to the pestilential vapors inhaled upon the *Chiaja*. A good system of sewerage, that would carry the filth of the city far out into the bay, with efficient police regulations,

* Formerly the rendezvous of Roman beggars, to keep streets clean, would make a great change in all this. At present, the sewerage is either upon the surface, or only slightly and imperfectly covered, and is discharged directly into the bay. The streets are terribly dirty, and the lower classes live in low, dark, damp rooms, reeking with filth. As a question of political economy, their lives are not worth much, and they are a source of constant anxiety to the government, but still they are human beings, and might be made to contribute largely to the wealth of the kingdom.

Rome, on its surface at least, is quite as filthy as Naples, but its sewerage is good; the *cloaca maxima*, constructed at an early period in the history of the city, is still perfect. The Tiber flows through the city with a strong impetuous current. Old aqueducts supply an abundance of pure water from the Alban hills. Almost every piazza has its fountain, which, day and night, winter and summer, sends forth its grateful streams. The population of Rome is about 200,000, occupying quite as much living space as the 500,000 of Naples.

The diseases of Rome are malarious. The zymotic forms or types are seldom seen, except as they are brought from other cities.

The campagna, as the country about Rome is called, is by no means a swamp, but is a wide plain, with but little cultivation, more like the prairies of Illinois than anything I have seen in Europe. During the spring and early summer, this prairie is covered with a rich, luxuriant vegetation, but, by July, a change begins to take place. The hot sun and south winds from, as the people here believe, the sands of Africa, convert these broad plains into a desert. With the sirocco comes the malignant forms of fever, just such as we see often in the Mississippi valley, a pernicious intermittent. In the city, they are less frequent and less severe than on the campagna, but Northern residents seldom remain after the 1st of July, or return before the 1st of October.

Florence is not only a beautiful, but also a healthy city.

"Girt by her theatre of hills, she reaps
Her corn, and wine, and oil, and plenty leaps
To laughing life with her redundant horn."

The "smiling Arno sweeps" rapidly through the city. The streets are clean, though, for the most part narrow; and the dwellings are high and, at the present time, very much crowded.

The fevers of Florence are of the continued form, but without intestinal complications, and not usually severe. I saw in the hospital, several characteristic cases of mild miliary fever.

Tubercular diseases are not uncommon throughout Italy, especially at Rome, and bronchitis and pneumonia are by no means rare. I think, in America, there is, very generally, an impression that the Italian winters, if not pleasant, are at least not disagreeable. It is true, they are not cold, as indicated by the thermometer, but they are damp, and most persons from the North experience more discomfort in Rome than they would in Edinburgh or Chicago. On the shores of the Mediterranean, at the feet of the Maritime Alps, are some very pleasant situations, which are comfortable during the winter. The moisture in the northerly winds is precipitated in passing over the mountains, while the sea breezes, if not dry, are warm. Nice, Monaca, and Genoa are so situated. From all I have learned, however, I should advise with extreme caution a winter residence in Italy. From November to March, unless it be in the extreme south, this is by no means a sunny clime; excepting, perhaps, as compared with England. The most favorable periods in which to visit the peninsula, are from the middle of March to the 1st of July, and the months of October and November. Its beauty of scenery and climate, during these periods can hardly be exaggerated.

Poets and painters have depicted the Italians as preëminently beautiful. Their descriptions apply only to childhood. Beauty is seldom retained beyond the period of puberty. They grow up without intellectual or moral culture, drink sour wines, live on salads and hard bread, seldom eat meat, and have no ambition, and no aims or purposes in life, beyond the gratification of physical appetites. I speak of the lower classes, and they constitute the great bulk of the population. It is, perhaps, almost impossible that, under such circumstances, any people can have a good physical development. The body is always, to some extent, moulded to the immortal shape created after God's own image. The *good*, the *beautiful*, and the *true* are inseparably connected.

In passing through Switzerland, I saw many cases of goitre and cretinism, for the most part, however, confined to the close valleys of the higher Alps. In other respects, the Swiss have a much better physical development than the Italians, perhaps because they have culture, freedom, self-respect, individuality. This little republic is the heart of Europe, beating strongly against its ribs of rock. In more senses than one, its pulsations are felt throughout the continent. Within its mountain fastnesses there are no beggars. Free schools, intelligence, industry everywhere. Almost every family owns its home, and every man is a sovereign. In crossing from Northern Italy, the contrast is very great. Long may the little republic flourish, the home of science, and a safe refuge for the oppressed from the surrounding despotisms.

I am under obligations to Dr. Jas. B. Gould, formerly surgeon in the U.S. Navy, but for several years in the practice of medicine in Rome, not only for many personal attentions, but also for much valuable information relating to public hygiene and medical matters in Italy. The Doctor is a man of fine professional attainments, and the most American of Americans.

Very truly, yours, H. A. JOHNSON.

---

### EXPULSION OF A FLESHY MASS FROM THE UTERUS.

Editor Chicago Medical Examiner:—Miss C., aged about 20 years, of light complexion, an American by birth, rather below the medium size, was quite unwell, having had intermittent fever. She had not menstruated for four months, and supposed she was pregnant. Her abdomen was enlarged, and I did not doubt but that she was pregnant. I heard no more from her, until the 10th of May, 1865, about five months after the occurrence above alluded to, when I was sent for in haste. When I arrived, she was suffering the most excruciating labor pains. About the time I was ready to make an examination, there was expelled a tumor (fleshy in texture), about four inches in length to three in thickness, without abating her sufferings. After examining the abdomen and making vaginal examination, I discovered no cause for such excruciating pain or suffering. The uterus was contracted, and smaller than after an ordinary labor, consequently I administered $\frac{1}{4}$ grain of morphia, repeating it in 30 minutes. Waiting 30 minutes, gave $\frac{1}{2}$ grain more, which relieved her and caused her to fall asleep. In one hour after, I took my leave, leaving her $\frac{1}{2}$ grain of morphia, to be given if her pains should return, which was given in three or four hours after, as her suffering was returning. I saw her next day, when she was quite comfortable, with the exception of some after-pains, discharging the lochia as in ordinary cases of confinement. From the time that she ceased to menstruate, until she was confined, she had very delicate health.

*June* 15*th*. I saw her to-day. She is pretty well, and has menstruated once. I report this case, not because I think it is very instructive, but because it is a case of rare occurrence, and, consequently, interesting. JNO. D. JENNINGS,

*Sonora, Ohio.*

